# IUC23530-78 Diagnostic value, safety, and patient-reported outcomes of total free-hand LATP biopsy technique

**DOI:** 10.1093/oncolo/oyaf248.005

**Published:** 2025-08-29

**Authors:** A Elftheriadou, F R Antara, C Collins, D Kannapiran, M Elhammadi, D D Carbin

**Affiliations:** Ashford and St Peter’s Hospitals NHS Foundation Trust, United Kingdom; Ashford and St Peter’s Hospitals NHS Foundation Trust, United Kingdom; Ashford and St Peter’s Hospitals NHS Foundation Trust, United Kingdom; Ashford and St Peter’s Hospitals NHS Foundation Trust, United Kingdom; Ashford and St Peter’s Hospitals NHS Foundation Trust, United Kingdom; Ashford and St Peter’s Hospitals NHS Foundation Trust, United Kingdom

## Abstract

**Background:**

Prostate biopsy is the key diagnostic modality for detecting prostate cancer, with the USS-guided transperineal approach being preferred over older techniques, performed under local (LATP) or general anesthesia. During LATP, trans perineal access systems can be used to stabilize the biopsy needle with the ultrasound probe. However, these devices are expensive, non-reusable, and may restrict clinicians, who often prefer the total free-hand approach (tF-LATP) for better access to prostatic zones. We aim to assess the pain tolerability, diagnostic value and safety of the tF-LATP technique.

**Methods:**

Patients undergoing tF-LATP for suspected prostate cancer from June 2022 to July 2024 were included, following informed consent. Data on PSA levels, prostate size, previous prostate mpMRI findings, cancer detection, need for further biopsy, tolerability and complications, and pain levels were collected.

**Results:**

Seventy-five patients (*n* = 75) underwent tF-LATP, with a median age of 67 years (IQR 11 years) and a mean age of 66.41 years (SD 6.98). Prostate size data were available for 66 patients, averaging 56.05 cc (SD 26.8). PSA levels mean of 22.72 ng/mL (SD 78.33). During the procedure, the VAS ranged from 1 to 4, with a mode of 2 and a mean of 2.08 (SD 0.71). Post-procedure VAS scores ranged from 1 to 2, with a mode of 1 and a mean of 1.05 (SD 0.23). There were no instances of urinary retention, sepsis or hematuria requiring admission, and no patients required re-biopsy for under-sampling or conversion to guided biopsies. The number of cores taken per patient had a median of 23 (range 11-42, IQR 10). The distribution of cores was as follows: 10-20 cores (*n* = 18), 20-30 cores (*n* = 37), 30-40 cores (*n* = 17), >40 cores (*n* = 2), and missing data for 1 patient. Cancer stage based on MRI was recorded for 67 patients as follows: T2a (*n* = 18), T2b (*n* = 8), T2c (*n* = 34), T3a (*n* = 3), and T3b (*n* = 4). Thirty-one (41.3%) patients had a negative biopsy, while 44 tested positive (58.7%) for prostate cancer.

**Conclusions:**

The tF-LATP technique demonstrates excellent safety, diagnostic efficacy, and satisfactory tolerability. With its cost-effectiveness and enhanced accessibility to all prostatic lobes, clinicians are encouraged to integrate this technique more widely into clinical practice to maximize its advantages.

